# Study of the Acute Toxicity of Scorpion *Leiurus macroctenus* Venom in Rats

**DOI:** 10.1155/2024/9746092

**Published:** 2024-07-17

**Authors:** Valery Gunas, Oleksandr Maievskyi, Nataliia Raksha, Tetiana Vovk, Oleksiy Savchuk, Serhii Shchypanskyi, Igor Gunas

**Affiliations:** ^1^ Department of Forensic Medicine and Law National Pirogov Memorial Medical University, Pyrohova Street, 56, Vinnytsia 21018, Ukraine; ^2^ Department of Clinical Medicine Educational and Scientific Center “Institute of Biology and Medicine” of Taras Shevchenko National University of Kyiv, Hlushkova Avenue, 2, Kyiv 03127, Ukraine; ^3^ Department of Biochemistry Educational and Scientific Center “Institute of Biology and Medicine” of Taras Shevchenko National University of Kyiv, Hlushkova Avenue, 2, Kyiv 03127, Ukraine; ^4^ Department of Human Anatomy National Pirogov Memorial Medical University, Pyrohova Street, 56, Vinnytsia 21018, Ukraine

## Abstract

**Background:**

The expansion of the territory of human habitation leads to inevitable interference in the natural range of distribution of one or another species of animals, some of which may be dangerous for human life. Scorpions—the Arachnida class and order Scorpiones—can be considered as such typical representatives. Scorpions of the Buthidae family pose a particular danger to humans. However, LD_50_ has not yet been defined for many species of this family, in particular, new representatives of the genus *Leiurus*. *Leiurus macroctenus* is a newly described species of scorpion distributed in Oman, and the toxicity of its venom is still unknown. Estimating the LD_50_ of the venom is the first and most important step in creating the antivenom and understanding the medical significance of the researched animal species. The purpose of this study was to determine the lethal dose (LD_100_), the maximum tolerated dose (LD_0_), and the average lethal dose (LD_50_) in rats when using *Leiurus macroctenus* scorpion venom.

**Methods and Results:**

15 sexually mature scorpions were used in the study, which were kept in the same conditions and milked by a common method (electric milking). For the study, 60 male rats were used, which were injected intramuscularly with 0.5 ml of venom solution with a gradual increase in the dose (5 groups, 10 rats in each), and 10 rats were injected intramuscularly with physiological solution as control group. LD calculations were done using probit analysis method in the modification of the method by V.B. Prozorovsky. The LD_0_ of *Leiurus macroctenus* scorpion venom under the conditions of intramuscular injection was 0.02 mg/kg, LD_100_ was 0.13 mg/kg, and LD_50_ was 0.08 ± 0.01 mg/kg.

**Conclusions:**

The analysis of scientific publications and other sources of information gives reason to believe that *Leiurus macroctenus* has one of the highest values of LD_50_ not only among scorpions but also among all arthropods in the world. All these point to the significant clinical importance of this species of scorpion and require further research that will concern the toxic effect of its venom on various organ systems. Determining the LD50 of the venom for new scorpion species is crucial for creating effective antivenoms and understanding the medical implications of envenomation by this species.

## 1. Introduction

Order Scorpiones has more than 2,400 species of scorpions, which is distinguished by the special structure of the telson with venomous glands, the danger of which ranges from local action (which is typical for about 95% of species) to serious consequences and death [1, 2]. The geographical distribution of scorpions is extremely wide and practically covers all parts of the land between the latitudes 50°N and 50°S, thus covering Europe, Asia, Africa, North and South America, Australia, and Oceania. The only continent completely free of scorpions is Antarctica.

This geographical distribution was reflected in the evolution of various species that ended up on different sides of the ocean, which in turn was reflected in the characteristics of the venom possessed by the scorpions of the New and Old World. Thus, for scorpions of the Old World, venoms with a predominance of *α*-toxins are more characteristic, while among scorpions of the New World, venoms based on *β*-toxins have become more common [3].

Epidemiological studies of scorpion stings in different countries are quite heterogeneous. Thus, if we take the Middle East, then there are no data from countries such as Syria, Yemen, Lebanon, or Bahrain. At the same time, a clear epidemiological situation is presented by Jordan, where 1,205 cases of bites were recorded in the period from 2006 to 2012, of which 2 were fatal, or in Saudi Arabia, 391 cases were detected in Riyadh province from 2006 to 2008, and Rabigh province recorded 41 cases, of which 1 was fatal in the period from 2007 to 2011 [4]. Data for 1 year of observation of only one hospital in Turkey revealed 52 records of scorpion bites in children, of which 1 ended fatally [5]. If we take into account data from other parts of the world, in Brazil more than 900,000 cases of scorpion bites were recorded, from 2000 to 2017, more than 1,000 of which resulted in the death of the patient [6]. In Australia, monitoring revealed 95 cases of scorpion bites from 2000 to 2002, none of which ended fatally [7].

Scorpion venom has significant toxicological and therapeutic importance due to its complex composition of bioactive molecules. Scorpion venom primarily contains neurotoxins that target ion channels in nerves and muscles, particularly sodium, potassium, calcium, and chloride channels. In addition to neurotoxins, it also contains small peptides, enzymes, mixtures of inorganic salts, free amino acids, nucleotides, amines, and lipids [8, 9]. All these elements in general lead to a systemic effect of venom on the entire body of the victim [8]. Understanding the mechanism of action of scorpion venom helps in developing antivenoms and improving clinical management of scorpion stings, which are a significant public health issue.

However, the data of many studies have shown that the elements of scorpion venom have significant therapeutic importance. Encouraging results are obtained in such areas as antimicrobial therapy where components of scorpion venom have shown activity against bacteria, fungi, and viruses, making them potential candidates for new antimicrobial agents; cancer therapy where some of venom components have demonstrated selective toxicity towards cancer cells, offering a basis for developing novel anti-cancer drugs; pain therapy where some venom components can block specific pain pathways without the addictive properties of conventional opioids; autoimmune and inflammatory diseases therapy where venom peptides are being investigated for their immunomodulatory properties, which could lead to new treatments for autoimmune diseases and chronic inflammatory conditions; and diseases of the cardiovascular system due to the participation of poison elements in the coagulation cascade [8, 10, 11].

Among all scorpion genera, the genus *Leiurus* has earned the most “venomous reputation,” namely, due to *Leiurus quinquestriatus*, which is the most venomous scorpion in the world to this day [12–15]. The venom of this genus is a powerful combination of neurotoxins [4, 16], chlorotoxins, and charybdotoxins [17]. The distribution area of this genus is quite extensive and includes the entire Middle East, north, east, and west [1, 18]. However, the volume of protein solution formed in the sting is quite small −0.225 mg [18]; therefore, most of the severe and fatal cases concern children.

Until the early 2000s, the diversity of the genus *Leiurus* was very limited, as a result of which taxonomy suffered and there was confusion in the identification of one or another species for decades. However, research conducted in the 21st century made it possible to bring order to the systematization of this genus and to discover a large number of new species in various countries of Africa and Asia, in particular on the Arabian Peninsula [18]. The *Leiurus macroctenus* species is one such recently described specie from the Buthidae family of the *Leiurus* genus. This scorpion species is common in some regions of Oman, and most of all it differs from its other relatives in the large size of the pectine teeth, which gave it the name “*macroctenus*” (i.e., large comb) [19]. Further genetic studies confirmed its existence as a separate species [1]. However, no research has yet been conducted to study the composition or toxicity of scorpion venom.

In particular, obtaining this knowledge will allow us to determine the amount of venom that can be fatal for a person. This is important for the development of appropriate safety measures and treatment in case of envenomation; also, the information obtained will help medical workers in developing and improving protocols for the treatment of scorpion envenomation. It can be used to determine the dose of antidote and other medicinal measures. In addition, the LD_50_ study contributes to the understanding of the effects of scorpion venom on different species of organisms, which can be important for biosecurity. Therefore, the determination of acute toxicity, which involves finding out the lethal dose (LD_100_), the maximum tolerated dose (LD_0_), and the average lethal dose of the venom, which causes the death of 50% of the animals of the group (LD_50_) in rats, became the aim of our study.

## 2. Materials and Methods

### 2.1. Sources of Scorpions and Rats

Based on the typical morphological characteristics of the species [19], *Leiurus macroctenus* scorpions were identified by Mark Stockmann and obtained from his private scorpion nursery in the city of Ibbenbüren (Germany). All scorpions were bred in captivity. For the study, 15 sexually mature individuals of both sexes were used, which were kept on a diet (1 *Shelfordella lateralis* cockroach per week), with access to water (weekly refilled distilled water), in plastic containers filled with sand (Exo Terra “Desert Sand”), with ventilation (reached by numerous holes in the boxes), constant temperature (25–35°C), humidity (25–35°C, 50–60% humidity), and natural lighting conditions. For at least a year, only cockroaches were used in food. In case of food refusal, the cockroach was removed from the container after 2 days. Once a month, the sand was freed from the remains of cockroaches.

The research used 70 white laboratory male rats grown in the vivarium of the Educational and Scientific Center “Institute of Biology and Medicine” of Taras Shevchenko Kyiv National University. Rats were kept on a standard diet in the conditions of an accredited vivarium in accordance with the “Standard Rules for the Arrangement, Equipment, and Maintenance of Experimental Biological Clinics (vivariums).” Experiments were conducted in accordance with the existing regulatory documents regulating the organization of work using experimental animals and compliance with the principles of the “European Convention on the Protection of Vertebrate Animals Used for Experimental and Other Scientific Purposes” [20]. Also, all work with animals was carried out in accordance with the Law of Ukraine dated February 21, 2006, No. 3447-IV “On the Protection of Animals from Cruelty” and in accordance with ethical norms and rules for working with laboratory animals [21].

The following conditions were observed in the room for keeping animals: temperature of 20–24°C, humidity of 30–70%, and 12-hour light day. Rats were fed standard food for laboratory animals. Experimental observations of animals were carried out during the working day from 9 : 00 a.m. to 6 : 00 p.m. The clinical condition of the experimental animals was monitored for 14 days. The appearance and development of clinical signs of envenomation and the timing of death or restoration of the body to normal were noted. When observing the manifestation of the toxic effect of venoms, attention was paid to the appearance of animals, their behavior, and their consumption of feed and water. After the death of the animals, a pathological autopsy was performed to detect macroscopic changes in organs and tissues.

### 2.2. Extraction of the Scorpion Venom

Milking of 15 sexually mature *Leiurus macroctenus* scorpions of both sexes was carried out once, according to Ozkan and Filazi's method [22], modified by Yaqoob et al. [23], 1 month after receipt from the nursery. Following the immobilization of the scorpion, the electrodes were directed towards the cephalothorax and telson. A 24 V electric current was then administered for 5 seconds to the base of the telson, while the opposite end of the telson was aimed towards the sterile vial. The amount of poison extracted during milking ranged from 0.1 to 0.5 mg. The venom was stored at a temperature of −20°C.

### 2.3. Grouping of Rats according to the Doses Administered

Rats that were selected for the experiment were subjected to a veterinary examination, after which they were divided into groups, weighed, numbered, and marked accordingly.

Animals weighing 200 g (±10 g) were randomly divided into 7 groups:

  Control group: 0.5 ml of physiological solution, which did not contain venom, was injected intramuscularly (in/m) (*n* = 10)
  Injected intramuscularly 0.5 ml of venom solution at a dose of 0.16 mg/kg (*n* = 10)
  0.5 ml of venom solution at a dose of 0.08 mg/kg (*n* = 10) was injected intramuscularly
  Injected intramuscularly 0.5 ml of venom solution at a dose of 0.06 mg/kg (*n* = 10)
  Injected intramuscularly 0.5 ml of venom solution at a dose of 0.04 mg/kg (*n* = 10)
  0.5 ml of venom solution at a dose of 0.02 mg/kg (*n* = 10) was injected intramuscularly
  0.5 ml of a venom solution at a dose of 0.01 mg/kg was injected intramuscularly (*n* = 10)

### 2.4. Administration of the Venom

The venom of scorpions from the family Buthidae, genus *Leiurus*, species *Leiurus macroctenus* was dissolved in 0.05 M Tris-HCl buffer (pH 7.4 + 0.13 M NaCl) and injected intramuscularly once.

Bibliosemantic analysis was used to select the dose. At the beginning of the experiment, we studied scientific literature data on previous toxicological studies. An analysis of the literature showed that the well-studied scorpion venom is the venom of scorpions from the family Buthidae of the genus *Leiurus* of the species *Leiurus quinquestriatus*. The LD_50_ of the venom for this species ranges from 0.16 to 0.50 mg/kg [12]. In connection with this, the first tested dose was 0.16 mg/kg, and the second was half of this dose −0.08 mg/kg, and so on.

### 2.5. LD Calculations

According to the results of the death of rats, LD_0_, LD_50_, LD_16_, LD_84_, and LD_100_, the error of LD_50_ was calculated by the method of probit analysis in the modification of method of V.B. Prozorovsky. Toxicometric parameters were calculated using the least squares method for probit analysis of lethality curves. The percentage of lethality, probits (Y), and weighting coefficients of probits (Z) is established. The formula of direct proportional dependence was used to construct the graph and calculate LD_50_ and its error [24].

The obtained results were processed by the methods of variational statistics using the StatPlus 5.9.8.5 software package and are presented in the form of average values with a standard deviation at the confidence level of 95%.

## 3. Results

It was established that there were no changes in the control rats, and when a dose of 0.16 mg/kg was used, all 10 rats died. In the group of animals with a dose of 0.08 mg/kg, 6 rats died and 4 rats were active and all changes returned to normal after 14 days. In groups of rats with a dose of 0.06 mg/kg, 3 rats died, and with a dose of 0.04 mg/kg, 1 rat died. Doses of 0.02 and 0.01 mg/kg did not cause death of animals.

When venom solutions are injected, a painful effect and numbness of the injected paw are observed. Swelling and redness of the eyes and nose were observed. During the first hour of the experiment, the development of inhibition, a decrease in response to external stimuli was registered and the rats lost coordination of movements, which was manifested in a shaky gait and falling on the side. In the first two days, almost complete rejection of food and water was recorded. The death of the animals was observed within the first two days after the introduction of the venom.

The next stage of studying the toxicological characteristics of the venom was the determination of the average lethal dose LD_50_ and its standard error, LD_16_, LD_84_, and LD_100_.

**Figure 1:**
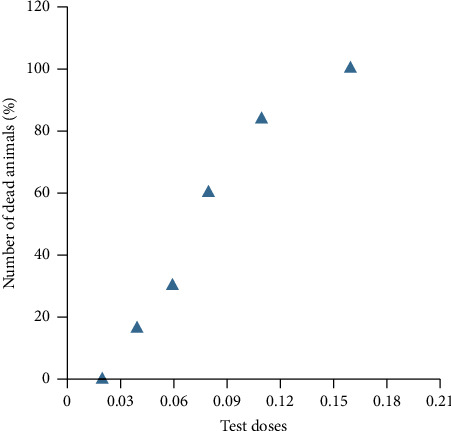
Parameters for determining LD_16_ and LD_84_ under the conditions of a single intramuscular injection of scorpion venom from the family Buthidae of the genus *Leiurus* of the species *Leiurus macroctenus* to rats.

As a result of the conducted studies, it was established that the maximum tolerant dose of *Leiurus macroctenus* scorpion venom (LD_0_—the smallest dose that does not cause death) under the conditions of intravenous administration is 0.02 mg/kg, the smallest lethal dose LD_16_ is 0.04 mg/kg, and the upper limit of lethality LD_84_ is 0.11 mg/kg (Figure 1). LD84/LD16 range of lethal doses (zone of acute toxic effect) is 0.04–0.11 mg/kg.

**Figure 2:**
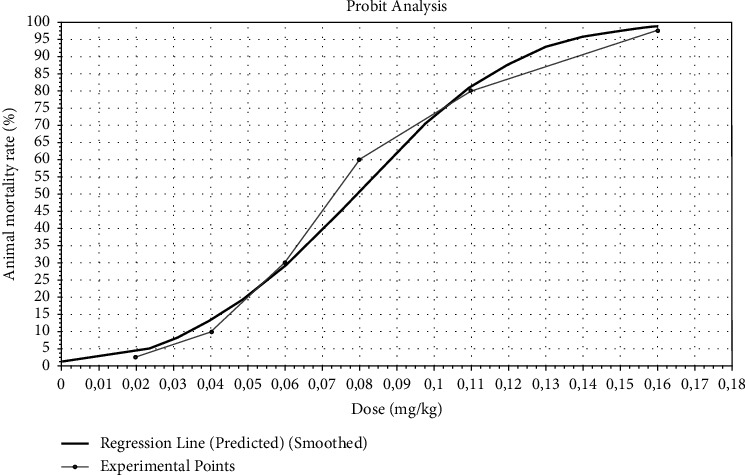
Parameters for determining LD_50_ under the conditions of a single intramuscular injection of scorpion venom from the family Buthidae of the genus *Leiurus* of the species *Leiurus macroctenus* to rats.

The LD_50_ is the dose of a toxin required to kill half the members of the test population in a given time period. LD_50_ is often used as a general indicator of the toxicity of a substance. The average lethal dose (LD_50_) was calculated using graphical probit analysis. Dose values (mg/kg) are plotted on the abscissa axis, and effect values (%) are plotted on the ordinate axis. A graphic representation of the curve characterizing the dose-effect relationship for experimental animals is shown in Figure 2.

The conducted studies allow us to determine the toxicological indicators of doses of venom of scorpions from the family Buthidae of the genus *Leiurus* of the species *Leiurus macroctenus* under the conditions of intramuscular administration: LD_50_ of 0.08 mg/kg, standard error of LD_50_ of 0.01; and LD_100_ of 0.13 mg/kg.  Table 1 summarizes the research results, data, and main indicators of the acute toxicity of *Leiurus macroctenus* scorpion venom, calculated using the Probit analysis method.

Thus, the LD_50_ of *Leiurus macroctenus* scorpion venom, given a single intramuscular injection to rats, is 0.08 ± 0.01 mg/kg of body weight, which allows it to be classified as an extremely toxic substance.

## 4. Discussion

Obtaining results for the first time on the values of LD_0_, LD_84_/LD_16_, LD_100_, and LD_50_ for the venom of *Leiurus macroctenus* requires a revision of the existing “hierarchy” of venom among scorpions. Considering the most common use of LD_50_ values in research and practice, it is considered as an indicator of toxicity.

Thus, the LD_50_ values established for three types of scorpions distributed in Iran *Hottentotta saulcyi*, *Hottentotta schach*, and *Androctonus crassicauda* were 1.47 mg/kg, 0.85 mg/kg, and 1.70 mg/kg, respectively. It is worth noting that, as in our study, the authors used male rats, but the injection was performed subcutaneously, which makes it difficult to compare the obtained data [25]. It is noteworthy that all three types of scorpions used in the work of Iranian scientists belong to the family Buthidae, which also includes the genus *Leiurus*, which is quite expected, since it is the family Buthidae that contains the largest number of scorpion species dangerous to humans.

*Hemiscorpius lepturus*, another representative of the Iranian fauna, in an experimental study on mice showed the values of LD_0_, 2.44; LD_50_, 6.33; and LD_100_, 11.71 mg/kg [26], but the method of venom administration was chosen intraperitoneally.

Among various representatives of the genus *Tityus*, the values of the sublethal dose range from 23.5 mg/kg for *T. falconensis* to 52.2 mg/kg for *T. Zulianus*. The *Tityus* family is a vivid representative of New World scorpions inhabiting Central and South America [27].

Until now, the lowest LD_50_ among scorpions belonged to *Leiurus quinquestriatus*, better known as the deathstalker, which is distributed in northern Africa and the Middle East. In an experiment on adult mice, which were injected with venom intraperitoneally, the values for females reached 0.09 mg/kg and for males 0.2 mg/kg [14]. According to data from other literary sources, the LD_50_ for this species of scorpion ranged from 0.25 to 0.33 mg/kg, which in any case were the lowest LD_50_ values among scorpions [13, 15]. Comparison of these data shows that *Leiurus macroctenus* can be named as the most venomous scorpion species on the planet, or as venomous as *Leiurus quinquestriatus* from this position with our data 0.08 ± 0.01 mg/kg for *Leiurus macroctenus*.

The closest values of sublethal doses (those with values less than 1 mg/kg) were found for scorpions of the genus *Androctonus*, namely, *A. oeneas oeneas*, *A. mauretanicus mauretanicus*, *A. crassicauda*, and *A. amoreuxi* with LD_50_ values of 0.31, 0.32, 0.40, and 0.75 mg/kg, respectively [13].

Most highly venomous scorpion species share certain morphological and behavioral aspects. Thus, the study of Van Der Meijden and coauthors [28] experimentally confirmed the widespread opinion that species with slender chelae and relatively large metasomas are more venomous. They also recorded that dangerously venomous scorpions (in particular, *Leiurus quinquestriatus*) most often use the telson for protection and rarely the chela. In addition, a correlation was found between high levels of LD_50_ and the presence of a long movable finger in a scorpion, which also fit well into the morphological description of *Leiurus* scorpions [19]. A similar opinion was reached by the team of authors of another study [29], who also associate long and thin chela with greater scorpion venom. In addition, the relationship between the size and the venomousness of the scorpion was found—the smaller the species is, the more venomous it is.

However, the results of an experiment to determine the venom of a scorpion depend on various and proven important factors that can either slightly reduce or increase the LD_50_ of the venom. All these factors require an understanding of the existence of inaccuracy in determining the semilethal of a venom and the need to provide as much information as possible in the “materials and methods” section.

One such factor is the “rest” period between milkings of the scorpion. On the example of *Centruroides limpidus*, it is shown that the recovery period of the venom volume is 5 days, and 13 days are needed to recover the total protein content and mammalian toxicity [30]. Such data are especially important for studies that use low-venom scorpion species (which require the accumulation of more venom) or with a limited number of scorpions for milking. To avoid this factor in our study, each scorpion was milked only once.

The influence of gender and duration of keeping in laboratory conditions on the composition of *Tityus* scorpion venom was proven by D'Suze and coauthors [27]. Their study provides data on changes in the composition of venom during the first 20 days of keeping scorpions, which was also taken into account when creating the model for this study. After receiving the scorpions, in our study, they were given a rest period of 1 month.

Manifestations of sexual dimorphism were also revealed during the study of *Centruroides vittatus*. In addition to the behavioral differences, manifested by the propensity of females to defend and males to flee from danger, differences in the composition of the venom were also revealed—the venom of males in the same amount as that of females caused more irritation in the mice that were injected with it [31]. All this is primarily related to the different sizes of the telson, the morphological structure, and, in particular, the difference in the ratio of cellular elements in males and [32]. Taking into account the data of the above works, this factor can be investigated by us in relation to *Leiurus macroctenus* in the following works.

Equally important is the nutrition factor of scorpions kept in laboratory conditions—which leads to changes in the qualitative and quantitative composition of the venom [33, 34]. When studying the influence of the diet on the amount of venom of *A. finitimus* and *H. tamulus*, it was established that the diet based on grasshopper nymphs significantly increased its amount compared to grasshopper adults, house crickets, moths, and house flies (*P* < 0.05 in both cases) [34]. This applies not only to scorpions but also to venomous snakes. In studies of adult female Dusky Pigmy rattlesnakes reared on different prey over a 26-month period, mice-fed individuals had a significant increase in the relative amounts of total PLA2 by 95% and serine proteinases by 100%, whereas lizard-fed females had the corresponding indicators of 42% and −22% and frogs of 2% and 11% [35]. Emma-Louise Davies and Kevin Arbuckle found evidence that a diet based on amphibians and reptiles in snakes is associated with neurotoxicity of venom [27].

Another factor that can both quantitatively and most importantly qualitatively affect the composition of the venom is the way the scorpion is milked. It is known for sure that the use of the electric method of milking is more effective than the manual method and allows obtaining not only statistically significantly larger amounts of venom but also a larger number of reproducible peaks, as evidenced by the data of Matrix-Assisted Laser Desorption Ionization Time of Flight Mass Spectrometry analysis [34]. These data were confirmed by Moroccan scientists when working with *Androctonus mauretanicus* and *Buthus occitanus tunetanus* [36].

As indicated in the above studies, intraperitoneal and intramuscular methods of venom administration are most often used, which raises the question of whether there is a difference in the assessment of LD_50_ results and, if so, which of them causes an increase in venom toxicity. There is no unequivocal answer to this question—yes, in a study using the venoms of four different species of venomous snakes, three of them showed an increase in toxicity with intraperitoneal injection and one showed the same toxicity both with intramuscular and intraperitoneal injection [37]. In addition, the homogeneity of the sample of experimental animals in terms of gender is important, since male and female mammalian experimental animals are differently sensitive to the effects of venom and, accordingly, have different LD_50_ values [38].

If we take ants into account, then the Argentine ant (*Linepithema humile*) shows the highest toxicity indicators with an LD_50_ value of 0.806–1.193 mg/kg. *D. bicolor* and *P. californicus* have slightly lower toxicity values with values of 2.42–3.36 and 3.42–4.92 mg/kg [39].

Certain species of centipedes are dangerous to humans and can cause death. Thus, the highest LD_50_ values were found in the variety *Otostigmus scabricauda* with a value of 12 mg/kg, respectively [40].

Among wasps in terms of toxicity, the greatest medical value is *Polistes olivaceus*, which has a semilethal value of 5.54 mg/kg [41].

Among the representatives of the order Araneae, the species named “widows” has retained the most infamous reputation. Thus, high LD_50_ values were found in *Latrodectus geometricus* (0.43 mg/kg), known as “brown at home,” *Latrodectus mactans* (1.39 mg/kg), known as “black widow,” and *Latrodectus tredecimguttatus* (0.59 mg/kg), known as “Mediterranean black widow” [42].

Even when compared with animals that do not belong to the phylum arthropods, the LD_50_ values of the scorpion we studied were higher than those of many species of snakes. For example, *Montivipera latifii*, 0.84 mg/kg; *Caucasicus intemedius Agkistrodon*, 1.45 mg/kg; *Vipera raddei*, 1.63 mg/kg; *Vipera albicornuta*, 2.05 mg/kg; *Macrovipera lebetina*, 3.87 mg/kg were administered intraperitoneally to mice [26]. For *Naja naja karachiensis*, commonly known as the Indian cobra, the LD_50_ intramuscularly is 1.2 mg/kg [43].

LD50 is defined as the average dose that kills 50% of the study population. Acute lethality is a simple generalized indicator of the toxicity of any substance that is used in various industries, but the main thing is that such an indicator is standardized and universal [44].

Leiurus is one of the genera of medical importance. Data on the effects of scorpion venom on humans show its effects on various organ systems. Thus, *Leiurus abdullahbayrami* venom causes convulsions, weakness, excitement, and aggressiveness. The venom of other scorpion species also has a pronounced effect on the human body: *Tityus pachyurus polock* venom causes ataxia, restlessness, drowsiness, hypoglycemia, salivation, and respiratory distress. *Tityus stigmurus* causes cardiogenic shock, pulmonary edema, and severe neurological symptoms [45].

Obtaining preliminary data on LD_50_ allows to further investigate the effectiveness of the use of various medicinal products, for example, to evaluate the preventive and therapeutic effects of drugs, which can be achieved by conducting experimental studies on animal models [46].

## 5. Conclusion

This study showed that the value of the LD_50_ dose of *Leiurus macroctenus* scorpion venom, for a single intramuscular injection to male rats, is 0.08 ± 0.01 mg/kg of body weight, which makes it one of the most venomous representatives not only among scorpions but also among other arthropods in the world, including spiders, scolopendras, and other representatives of this phylum.

The results of this study showed that the investigated scorpion has a therapeutic value and therefore requires further research, in particular the study of the effect on various internal organs.

The obtained data can be successfully applied in the future to interpret what amount of venom can be fatal for a person, which in turn can be used to develop treatment methods, in particular, to estimate the necessary dose of antivenom.

## Figures and Tables

**Table 1:** Parameters of the acute toxicity of scorpion venom from the family Buthidae of the genus *Leiurus* of the species *Leiurus macroctenus* on rats.

Dose (mg/kg)	Number of dead animals	Lethality (%)	Effect in probits y	“Weight” factor punched Z
0.01	0	0	3.04	1.0
0.02	0	0	3.04	1.0
0.04	1	16	3.72	2.6
0.06	3	30	4.48	4.5
0.08	6	60	5.25	4.7
0.11	8	84	5.84	3.9
0.16	10	100	6.96	1.2

## Data Availability

The data that support the findings of this study are available from the corresponding author upon reasonable request.

## References

[1] Alqahtani A. R., Badry A. (2020). Genetic diversity among different species of the genus Leiurus (Scorpiones: Buthidae) in Saudi Arabia and the Middle East. *Saudi Journal of Biological Sciences*.

[2] Howard R. J., Edgecombe G. D., Legg D. A., Pisani D., Lozano-Fernandez J. (2019). Exploring the evolution and terrestrialization of scorpions (Arachnida: Scorpiones) with rocks and clocks. *Organisms, Diversity and Evolution*.

[3] Abroug F., Ouanes-Besbes L., Tilouche N., Elatrous S. (2020). Scorpion envenomation: state of the art. *Intensive Care Medicine*.

[4] Amr Z. S., Baker M. A., Al-Saraireh M., Warrell D. A. (2021). Scorpions and scorpion sting envenoming (scorpionism) in the Arab Countries of the Middle East. *Toxicon*.

[5] Bosnak M., Ece A., Yolbas I., Bosnak V., Kaplan M., Gurkan F. (2009). Scorpion sting envenomation in children in southeast Turkey. *Wilderness and environmental medicine*.

[6] Monteiro W. M., Gomes J., Fé N. (2019). Perspectives and recommendations towards evidence-based health care for scorpion sting envenoming in the Brazilian Amazon: a comprehensive review. *Toxicon*.

[7] Isbister G. K., Volschenk E. S., Balit C. R., Harvey M. S. (2003). Australian scorpion stings: a prospective study of definite stings. *Toxicon*.

[8] Ahmadi S., Knerr J. M., Argemi L. (2020). Scorpion venom: detriments and benefits. *Biomedicines*.

[9] Tobassum S., Tahir H. M., Arshad M., Zahid M. T., Ali S., Ahsan M. M. (2020). Nature and applications of scorpion venom: an overview. *Toxin Reviews*.

[10] Ghosh A., Roy R., Nandi M., Mukhopadhyay A. (2019). Scorpion venom–toxins that aid in drug development: a review. *International Journal of Peptide Research and Therapeutics*.

[11] Cid-Uribe J. I., Veytia-Bucheli J. I., Romero-Gutierrez T., Ortiz E., Possani L. D. (2020). Scorpion venomics: a 2019 overview. *Expert Review of Proteomics*.

[12] Alqahtani A. R., Badry A. (2020). Genetic diversity among different species of the genus Leiurus (Scorpiones: Buthidae) in Saudi Arabia and the Middle East. *Saudi Journal of Biological Sciences*.

[13] Habermehl G. (2012). *Venomous Animals and Their Toxins*.

[14] Salama W. M. (2013). The lethal effect of leiurus quinquestriatus venom on adult and weanling albino mice with evaluating its effects on some biochemical parameters. *Egyptian Journal of Zoology*.

[15] Van der Meijden A., Koch B., Van der Valk T., Vargas-Muñoz L. J., Estrada-Gómez S. (2017). Target-specificity in scorpions; comparing lethality of scorpion venoms across arthropods and vertebrates. *Toxins*.

[16] Zilberberg N., Zlotkin E., Gurevitz M. (1992). Molecular analysis of cDNA and the transcript encoding the depressant insect selective neurotoxin of the scorpion Leiurus quinquestriatus hebraeus. *Insect Biochemistry and Molecular Biology*.

[17] Erdeş E., Doğan T. S., Coşar İ (2014). Characterization of Leiurus abdullahbayrami (Scorpiones: Buthidae) venom: peptide profile, cytotoxicity and antimicrobial activity. *Journal of Venomous Animals and Toxins including Tropical Diseases*.

[18] Lourenço W. R. (2020). Why does the number of dangerous species of scorpions increase? The particular case of the genus Leiurus Ehrenberg (Buthidae) in Africa. *Journal of Venomous Animals and Toxins including Tropical Diseases*.

[19] Lowe G., Yağmur E., Kovařík F. (2014). A review of the genus Leiurus Ehrenberg, 1828 (Scorpiones: Buthidae) with description of four new species from the Arabian Peninsula. *Euscorpius*.

[20] Louhimies S. (2002). Directive 86/609/EEC on the protection of animals used for experimental and other scientific purposes. *Alternatives to Laboratory Animals*.

[21] On the protection of animals from cruel treatment (2006). The law of Ukraine dated 21.02. https://zakon.rada.gov.ua/laws/show/3447-15#Text.

[22] Ozkan O., Filazi A. (2004). The determination of acute lethal dose-50 (LD50) levels of venom in mice, obtained by different methods from scorpions, Androctonus crassicauda (Oliver 1807). *Acta Parasitologica*.

[23] Yaqoob R., Tahir H. M., Arshad M., Naseem S., Ahsan M. M. (2016). Optimization of the conditions for maximum recovery of venom from scorpions by electrical stimulation. *Pakistan Journal of Zoology*.

[24] Prozorovsky V. B. (2007). Statistical processing of the results of pharmacological studies. *Psychopharmacol Biol. Narcol.*.

[25] Boghozian A., Nazem H., Fazilati M., Hejazi S. H., Sheikh S. M. (2021). Toxicity and protein composition of venoms of Hottentotta saulcyi, Hottentotta schach and Androctonus crassicauda, three scorpion species collected in Iran. *Veterinary Medicine and Science*.

[26] Fathi B., Younesi F., Salami F. (2022). Acute venom toxicity determinations for five Iranian vipers and a scorpion. *Iranian Journal of Toxicology*.

[27] D’Suze G., Sandoval M., Sevcik C. (2015). Characterizing Tityus discrepans scorpion venom from a fractal perspective: venom complexity, effects of captivity, sexual dimorphism, differences among species. *Toxicon*.

[28] Van Der Meijden A., Lobo Coelho P., Sousa P., Herrel A. (2013). Choose your weapon: defensive behavior is associated with morphology and performance in scorpions. *PLoS One*.

[29] Forde A., Jacobsen A., Dugon M. M., Healy K. (2022). Scorpion species with smaller body sizes and narrower chelae have the highest venom potency. *Toxins*.

[30] Carcamo-Noriega E. N., Possani L. D., Ortiz E. (2019). Venom content and toxicity regeneration after venom gland depletion by electrostimulation in the scorpion Centruroides limpidus. *Toxicon*.

[31] Miller D. W., Jones A. D., Goldston J. S., Rowe M. P., Rowe A. H. (2016). Sex differences in defensive behavior and venom of the striped bark scorpion Centruroides vittatus (Scorpiones: Buthidae). *Integrative and Comparative Biology*.

[32] Sentenská L., Graber F., Richard M., Kropf C. (2017). Sexual dimorphism in venom gland morphology in a sexually stinging scorpion. *Biological Journal of the Linnean Society*.

[33] Gangur A. N., Smout M., Liddell M. J., Seymour J. E., Wilson D., Northfield T. D. (2017). Changes in predator exposure, but not in diet, induce phenotypic plasticity in scorpion venom. *Proceedings of the Royal Society B: Biological Sciences*.

[34] Tobassum S., Tahir H. M., Zahid M. T., Gardner Q. A., Ahsan M. M. (2018). Effect of milking method, diet, and temperature on venom production in scorpions. *Journal of Insect Science*.

[35] Gibbs H. L., Sanz L., Chiucchi J. E., Farrell T. M., Calvete J. J. (2011). Proteomic analysis of ontogenetic and diet-related changes in venom composition of juvenile and adult Dusky Pigmy rattlesnakes (Sistrurus miliarius barbouri). *Journal of Proteomics*.

[36] Oukkache N., Chgoury F., Lalaoui M., Cano A. A., Ghalim N. (2013). Comparison between two methods of scorpion venom milking in Morocco. *Journal of Venomous Animals and Toxins including Tropical Diseases*.

[37] Oukkache N., Jaoudi R. E., Ghalim N. (2014). Evaluation of the lethal potency of scorpion and snake venoms and comparison between intraperitoneal and intravenous injection routes. *Toxins*.

[38] Pucca M. B., Roncolato E. C., Campos L. B. (2011). Experimental Tityus serrulatus scorpion envenomation: age-and sex-related differences in symptoms and mortality in mice. *Journal of Venomous Animals and Toxins including Tropical Diseases*.

[39] Greenberg L., Kabashima J. N., Allison C. J. (2008). Lethality of red imported fire ant venom to Argentine ants and other ant species. *Annals of the Entomological Society of America*.

[40] Han Y., Kamau P. M., Lai R., Luo L. (2022). Bioactive peptides and proteins from centipede venoms. *Molecules*.

[41] Liu H., Gao P., Wu X. (2012). Utilization of Polislidae wasp venom as potential new insect drugs in the R&D of wellness industry. *International Journal of Biotechnology for Wellness Industries*.

[42] Rauber A. (1983). Black widow spider bites. *Journal of Toxicology-Clinical Toxicology*.

[43] Riaz Z., Zaman M. Q., Ullah Z. (2015). Bio-physiological effects of LD50 of crude venom of black Pakistani cobra (Naja naja karachiensis) in mice. *J. Anim. Plant. Sci.*.

[44] Morris-Schaffer K., McCoy M. J. (2020). A review of the LD50 and its current role in hazard communication. *ACS Chemical Health and Safety*.

[45] Saganuwan S. A. (2021). Toxicosis of snake, scorpion, honeybee, spider, and wasp venoms: Part 2. InMedical. *Toxicology*.

[46] Guieu R., Kopeyan C., Rochat H. (1993). Utilization of aspirin, quinine and verapamil in the prevention and treatment of scorpion venom intoxication. *Life Sciences*.

[47] Davies E. L., Arbuckle K. (2019). Coevolution of snake venom toxic activities and diet: evidence that ecological generalism favours toxicological diversity. *Toxins*.

